# Transient Central Diabetes Insipidus after Discontinuation of Vasopressin

**DOI:** 10.1155/2019/4189525

**Published:** 2019-12-11

**Authors:** Nathaniel Carman, Carl Kay, Abigail Petersen, Maria Kravchenko, Joshua Tate

**Affiliations:** ^1^Department of Medicine, Brooke Army Medical Center, Fort Sam Houston, TX, USA; ^2^Uniformed Services University of the Health Sciences, Bethesda, MD, USA

## Abstract

Central diabetes insipidus (CDI) is an uncommon condition resulting from lack of vasopressin secretion from the posterior pituitary gland typically caused by some form of destruction of the gland. Here we present a case of transient CDI after discontinuation of vasopressin used for septic shock without evidence of overt pituitary damage. Serum sodium concentration peaked at 160 mmol/L in the setting of polyuria within days of vasopressin discontinuation without identified alternative etiologies. Sodium levels and urine output normalized with administration of desmopressin with continued stability after desmopressin was discontinued. This is one of few reported cases of diabetes insipidus occurring after discontinuation of vasopressin and the rapid and profound response to desmopressin in this case proves a central etiology. This case allows for speculation into predisposing risk factors for this phenomenon including preexisting neurological disease.

## 1. Introduction

Central diabetes insipidus (CDI) is characterized by deficient secretion of vasopressin, a hormone produced in the hypothalamus and secreted from the posterior pituitary gland. The lack of vasopressin leads to hallmark findings of polyuria, polydipsia and hypernatremia. The most common and well described causes of CDI are secondary to injury and destruction of the hypothalamus or posterior pituitary including trauma, neurosurgery, tumors, ischemia and autoimmunity [1]. What is less well described is CDI following cessation of therapeutic exogenous vasopressin, a common treatment for patients with vasodilatory shock and refractory hypotension. Few cases of CDI related to withdrawal of vasopressin have been reported with the actual incidence of vasopressin associated CDI remaining largely unknown [2–6]. While sparse in the literature, there have been reports of this phenomenon allowing for speculation of potential predisposing risk factors as well as underlying mechanisms. We report a case of transient CDI following discontinuation of vasopressin for treatment of urinary tract infection-induced septic shock.

## 2. Case Presentation

**Figure 1:**
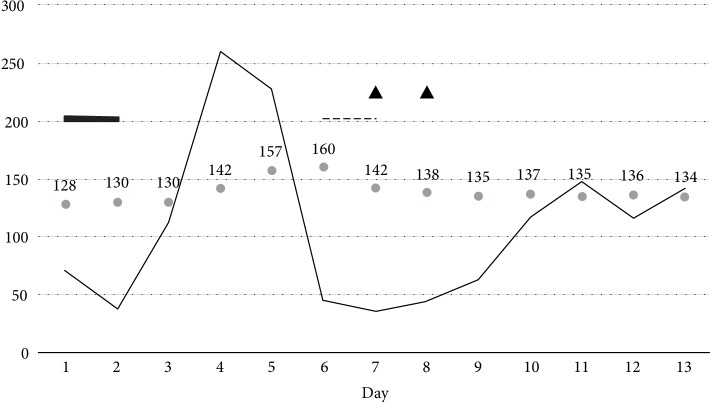
Overview of serum sodium and urine output trends in relation to therapies. Thin solid line represents average urine output (mL/hr), numbered bullet points showing measured serum sodium (mmol/L), thick solid line denoting time of intravenous vasopressin administration, dotted line denoting period of intravenous desmopressin administration, and triangles show intranasal desmopressin administration.

An 88-year-old male with a history of normal pressure hydrocephalus and ventriculoperitoneal (VP) shunt presented to the emergency department for altered mental status, fever, and hypotension. Evaluation was notable for hyponatremia [serum sodium 129 mmol/L (reference range 133–145 mmol/L)] and *Pseudomonasaeruginosa* positive urine culture. Septic shock was treated via volume resuscitation, vasopressors, and broad-spectrum antibiotics. Vasopressors, norepinephrine and vasopressin (0.04 units/min), were weaned off approximately 24 hours after intensive care unit admission (Figure 1). From hospital days 3–5, the patient experienced an acute rise in serum sodium concentration (130 mmol/L–159 mmol/L) associated with a diuresis of 18 liters over 72 hours (average output of 250 mL/hr). The patient received no diuretics and serum glucose was without hyperglycemic excursions while on a subcutaneous insulin regimen. Serum osmolality was 344 (285–310) mOsm/kg and urine osmolality was 203 (300–1300) mOsm/kg with rise to 545 mOsm/kg after administration of desmopressin 2 mcg intravenously. A diagnosis of CDI was confirmed. Computed tomography of the head showed stable placement of the VP shunt and no acute abnormalities. Magnetic resonance imaging was unobtainable due to presence of a pacemaker. Concomitant adrenal insufficiency was ruled out with a cosyntropin stimulation test. Urine output fell to <1.5 L/day after initiation of desmopressin therapy. Serum sodium concentration was initially unresponsive with maximum value of 160 mmol/L falling to normal after addition of intravenous infusion of 5% dextrose in water. Desmopressin 4 mcg twice daily was continued for 24 hours. On hospital day 7, the intravenous 5% dextrose in water solution infusion was discontinued and patient transitioned to once daily intranasal administration of desmopressin, with recurrence of mild hyponatremia. Desmopressin administration was discontinued on hospital day 8, with the sodium concentration and urine output remaining normal during the remainder of the hospital stay. To present, the patient has had no recurrence of polyuria or symptoms of hypernatremia; however, he elected to discharge home on hospice care with no available follow-up serum sodium testing.

## 3. Discussion

Vasopressin is a commonly used agent in intensive care units for treatment of vasodilatory shock. A sparsely reported adverse effect of vasopressin is rebound polyuria and hypernatremia upon discontinuation consistent with diabetes insipidus [7]. Moreover, there are very few case reports of transient diabetes insipidus (tDI) associated with vasopressin discontinuation in the absence of other well-described causes. With concerns that this may be an underreported and underappreciated occurrence, it becomes increasingly important to identify potential risk factors for transient vasopressin-related CDI and potential mechanisms.

One confounding factor in the current literature of a vasopressin-withdrawal-induced DI is the high incidence of concomitant disease processes known to independently cause central DI. It is well documented that subarachnoid hemorrhage (SAH) is a common etiology of transient central diabetes insipidus, being observed in 15% of all cases of SAH [8]. Five of six patients reported to have tDI in Bohl et al. had acute SAH [9]. However, the observed tDI was still attributed to withdrawal of vasopressin.

Excluding cases with these confounding factors, we are able to speculate on possible risk factors that predispose to the development of tDI after discontinuation of vasopressin based on similarities of individual reported cases [2–6]. As seen in Table 1, the previously reported cases were seen in five male and one female patient. Age is typically less than 65 years old; however, our case is notably older than other cases reported. Three cases reported a prior diagnosis of hydrocephalus, with two having a VP shunt, and two cases reported a remote history of intracranial bleed. Albeit from a small sample size, it can be posited that a potential predisposing condition to development of this phenomenon is preexisting neurological disease. Excluding cases with strong alternative causes of tDI as mentioned above, septic shock was the initial indication for vasopressin therapy in all cases. Notably, one reported case of tDI occurred after severe sepsis without initial vasopressin exposure. This may suggest an independent role of inflammatory cytokines in the development of tDI [10].

Previously proposed mechanisms for tDI after vasopressin withdrawal are numerous and include depletion of endogenous stores, antibody-mediated competitive inhibition of vasopressin, negative feedback repressing vasopressin production and release, and down regulation of the V2 receptor for vasopressin in the nephron causing a nephrogenic DI [3, 4, 9]. It has also been suggested that idiopathic CDI may be associated with abnormal blood supply to the posterior pituitary gland caused by vascular impairment of the inferior hypophyseal artery system, mechanisms relevant to our patient in the setting of hydrocephalus and septic shock that predispose to hypoperfusion [11].

Our patient's quick and profound response to desmopressin proved a central cause of DI. Previous studies show that endogenous vasopressin secretion would be markedly stimulated at a plasma osmolality of 344 mOsm/kg, as seen in our patient [1]. In a patient with a functional pituitary gland, plasma vasopressin concentration would be expected to exceed 10 pmol/L. Vasopressin's maximum antidiuretic effect occurs near plasma concentrations of 5 pmol/L [1, 3]. Our patient's drastic decrease in urine output and greater than a 2.5 times increase in urine osmolality after desmopressin administration suggests a deficiency in endogenous vasopressin. This case shows a clear central etiology of the tDI associated with vasopressin withdrawal as evidenced by our patient and likely similar cases. We favor a mechanism of depletion of endogenous hormone or negative feedback from exogenous vasopressin causing slowed production and release. These mechanisms may in part be related to the previously mentioned vascular impairment and inadequate blood supply to the posterior pituitary gland in the setting of acute illness.

In conclusion, we present a case of transient central diabetes insipidus following discontinuation of vasopressin therapy that provides further insight into potential risk factors for development of this phenomenon as well as the possible underlying mechanisms of its development. Future research should be directed at definitively investigating the underlying mechanism as well as identifying the true incidence and risk factors predisposing to the development of a vasopressin-withdrawal-associated tDI to identify those at highest risk.

## Figures and Tables

**Table 1:** Characteristics of reported cases of diabetes insipidus after vasopressin withdrawal. ^∗^Defined by rise of urine volume correlating with spike in Na through normalization of the values.

Case #	Age/sex	Duration of DI^∗^ (days)	Duration of vasopressin prior to DI (days)	Peak Na (mmol/L)	Relevant history	Reference
1	53/M	4	7	157	Hydrocephalus, VP shunt, SIADH, Pneumonia, Bacteremia, UTI	Kristeller (2004)
2	34/M	17	3	171	OHS, Pneumonia, Fungemia	Ramers (2005)
3	32/M	4	4	144	AAA, PFO, Mitral valve repair, Marfan's syndrome	Peskey (2009)
4	30/F	3	2	154	Tetrapelegia, Ruptured cervical AVM	Sundar (2016)
5	54/M	11	4	179	Down syndrome; Hypothyroidism, CVA, Pneumonia	Rana (2018)
6	88/M	3	1	160	Septic shock, Hydrocephalus, VP shunt	Garrahy (2019)

VP, ventriculoperitoneal; SIADH, syndrome of inappropriate antidiuretic hormone; UTI, urinary tract infection; OHS, obesity hypoventilation syndrome; AAA, abdominal aortic aneurysm; PFO, patent foramen ovale; AVM, arteriovenous malformation; CVA, cerebrovascular accident.
